# The Incidence of New Vertebral Fractures Following Vertebral Augmentation: A Meta-Analysis of Randomized Controlled Trials

**DOI:** 10.1097/MD.0000000000001532

**Published:** 2015-09-18

**Authors:** Weixing Xie, Daxiang Jin, Chao Wan, Jinyong Ding, Shuncong Zhang, Xiaobing Jiang, Jixi Xu

**Affiliations:** From the Department of Orthopaedics, The First Affiliated Hospital of Guangzhou University of Traditional Chinese Medicine, Baiyun District, Guang Zhou City, Guangdong Province, China.

## Abstract

A meta-analysis of randomized controlled trials was performed that compares the relationship between percutaneous vertebral augmentation (PVA) and conservative treatments with the incidence of new vertebral fractures.

Using meta-analytic techniques, this study compares PVA and conservative treatment for incidence of new vertebral fractures, particularly incidence of adjacent fractures that occur following treatment.

A focus of clinicians has been on whether PVA increases the risk of new vertebral fractures.

Pubmed, EMBASE, and the Cochrane Central Register of Controlled Trials were searched to retrieve literature published from the establishment of the databases until April 28, 2015. Literature of related areas was searched manually. The main outcome indicator was the incidence of new vertebral fractures at final follow-up appointment. In addition, we evaluated the incidence of new vertebral fractures in different follow-up periods and the incidence of adjacent fractures. The RevMan 5.3 software program of the Cochrane Collaboration was used to analyze the data. For dichotomous variables, the risk ratio (RR) and a confidence interval (CI) of 95% were used to express the heterogeneity of the effect size.

Seven randomized controlled trial studies were selected from the literature. The studies include 871 patients, 436 of whom received PVA treatment and the rest received conservative treatment. Combined analysis of the 7 studies showed that the numbers of new vertebral fractures in the 2 groups are not significantly different. Six studies reported the numbers of new adjacent fractures. Considering the heterogeneity among the studies, 2 subgroups were formed. The 5 studies in the European group showed that the incidence of new adjacent fractures in the PVA-treated group is higher than that in the conservatively treated group, and the difference is statistically significant. The one study in the Asian group showed no significant difference between the incidences of adjacent fractures in the 2 groups.

PVA treatment does not increase the incidence of new vertebral fractures. Most studies reported that PVA increases the incidence of adjacent fractures, yet it is rarely stated that both PVA and conservative treatment lead to the same incidence of adjacent fractures.

## INTRODUCTION

Percutaneous vertebral augmentation (PVA) refers to percutaneous vertebroplasty (PVP) and percutaneous kyphoplasty (PKP). It is a minimally invasive technique. By injecting bone cement into fractured vertebrae via percutaneous procedure, the treatment rapidly relieves the pain of patient, restore vertebral height partially, and provide biomechanical stability. Even though PVA is widely used to treat painful osteoporotic vertebral compressive factures, there are still many complications associated with this technique, such as the development of vertebral fractures following treatment.

Although it has been reported that PVA treatment could increase the incidence of new vertebral fractures, especially adjacent fractures,^1^ no strong positive connections between PVA treatment and the development of new vertebral fractures have been confirmed. Some studies have shown that patients that received PVA treatments have higher chances of suffering from re-fractures, and the development of re-fractures occur sooner than with patients who received conservative treatment. However, some scholars believe that the development of new fractures is a natural process associated with osteoporosis,^2^ and that PVA treatment does not increase the incidence of new vertebral fractures.

This article uses a meta-analysis to examine the relationships between controlled trials of PVA treatments and conservative treatments to vertebral compressive fractures. The analysis aims to evaluate the overall incidence of new vertebral fractures, time to new fractures, and incidence of adjacent fractures after PVA or a conservative treatment.

## SEARCH STRATEGY AND SELECTION CRITERIA

Pubmed, EMBASE, and the Cochrane Central Register of Controlled Trials were searched to retrieve English literature from the establishment of the databases until April 28, 2015. The keywords for the study object (MeSH words or free words) included “vertebral compression fractures” and “osteoporosis.” For the intervention strategy, the keywords were “vertebroplasty,” “kyphoplasty,” and “vertebral augmentation.” For post-treatment observations, the keywords were “new vertebral fractures,” “secondary vertebral fractures,” “subsequent vertebral fractures,” “adjacent vertebral fractures,” “refractures,” and “worsening.” Literatures of related areas were manually searched and the literature referenced by the selected articles was also checked. When required, the authors of the articles were contacted. All analyses were based on previous published studies; thus, no ethical approval and patient consent are required.

The following criteria were used to select a study for our meta-analysis. First, the study must be conducted through a randomized controlled trial (RCT). Second, the intervened subjects were patients suffering from osteoporotic vertebral compressive fractures. Third, the study was a comparative study between patients who received PVA (PVP or PKP) treatments and patients in the control group who received nonoperative treatment (patients who received conservative treatment or pseudo-operative treatment). Fourth, the observation index included the incidence or time of new vertebral fractures after treatment. Fifth, the article must be written in English.

The studies were excluded from our meta-analysis if they were not conducted through a randomized controlled trial; the intervention strategy or control group settings were not in accordance with our selection criteria; or they had repeated data to another study.

## QUALITY ASSESSMENT

Two evaluators independently selected studies, extracted data, performed methodological quality evaluations and then cross-validated the results. When disagreement occurred between the 2 evaluators, a third evaluator was involved. The Cochrane Collaboration risk assessment tool was used to evaluate the level of bias involved in this study. The RCT bias risk was assessed according to the correctness of the randomization; whether the grouping is confined and correct; whether blinding is adopted; completeness of the results; whether the study results are reported selectively; and whether other potential bias exists. For each criterion, an assessment of “low-degree bias,” “unclear,” or “high-degree bias” was given.

## OUTCOME VARIABLES

The primary outcome indicator is the incidence of new vertebral fractures at final follow-up. As it has been reported^1^ that PVA may accelerate the development of new vertebral fractures and increase the chance of adjacent fractures, we also evaluated the incidence of new vertebral fractures in different follow-up periods (within 3 months or over 1 year) and the incidence of adjacent fractures.

## META-ANALYSIS METHODS

The RevMan5.3 software program of the Cochrane Collaboration was employed to analyze the data. First, the *Q*-test and *I*^*2*^ value calculations were adopted to analyze the heterogeneity of the data. When the *P* value is <0.1 and the *F* value >50%, a greater level of heterogeneity exists among the selected studies. If there is no heterogeneity, a fixed-effects model was used for analysis. Otherwise, the cause of heterogeneity was first analyzed to decide whether a random-effects model could be used. For dichotomous variables, the risk ratio (RR) and the confidence interval (CI) of 95% were used to express the heterogeneity of effect size.

## RESULTS

### Literature Selection Results

**FIGURE 1:**
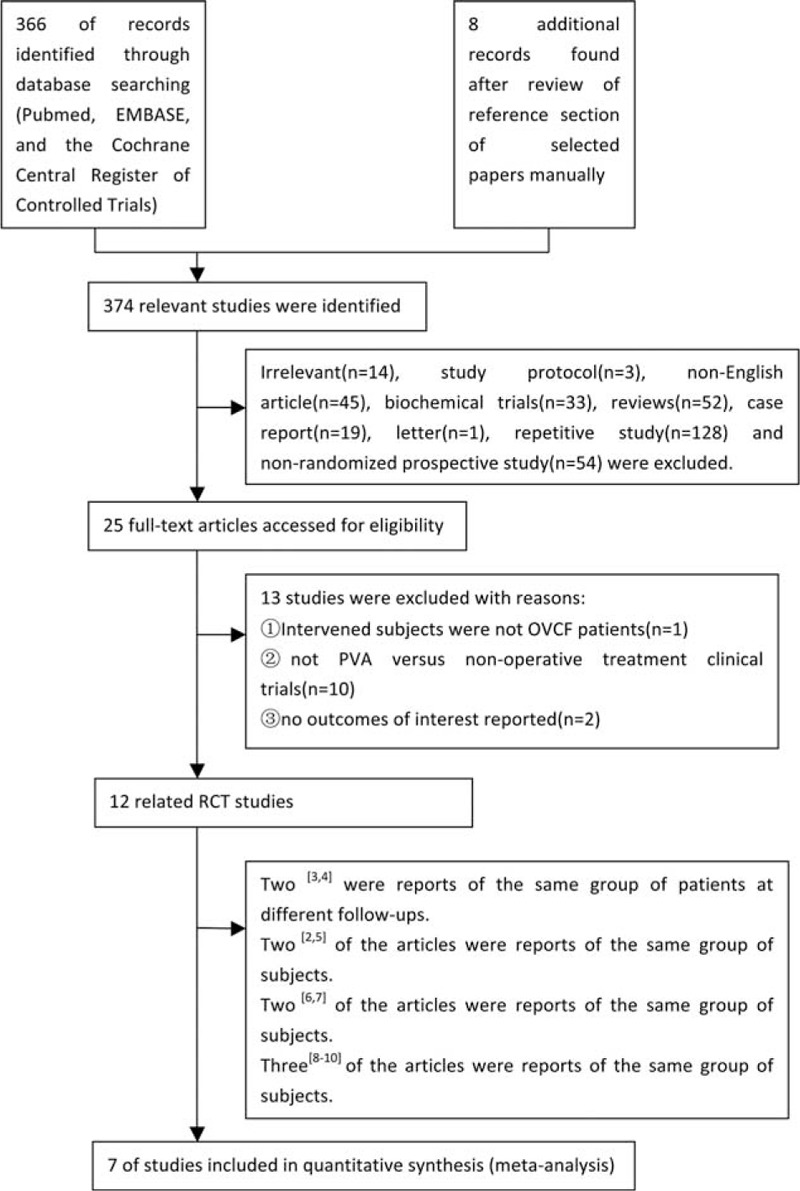
Literature search methodology.

The initial search of literature produced 374 articles of interest. After scanning the abstracts, 79 prospective studies were selected. Two reviewers carefully read those 79 articles and excluded 54 non-RCT studies, as well as 13 RCT studies that did not fit the selection criteria. In total, 12 RCT studies that satisfied the selection criteria were identified. Among those 12 RCT studies, 2^3,4^ were reports of the same group of patients at different follow-ups. Thus, the study of the later follow-up was chosen. In addition, 2,^2,5^ 2,^6,7^ and 3^8–10^ of the articles were reports of the same group of subjects, respectively. Thus, each of those with the more complete dataset of new vertebral fractures was selected. The literature selection process is illustrated in Figure 1. In the end, 7 articles^3,5,6,8,11–13^ were selected.

### Study Characteristics

Tables 1 and 2 present the basic information of the 7 selected RCT studies. The articles were published between 2007 and 2013. Each study involved 34 to 300 patients, adding up to a total of 871 patients, including 436 PVA-treated patients and 435 conservatively treated patients. Six of the 7 studies adopted PVP as an intervention strategy, and the others adopted PKP. The follow-up time ranged from 2 weeks to 24 months. Four^3,8,11,12^ of the studies reported the number of new vertebral fractures within 3 months of treatment, as shown in Table 3. Four^3,6,8,13^ of the studies reported the number of new vertebral fractures over 1 year, as shown in Table 4. Six^3,5,6,8,11,13^ of the studies reported the number of adjacent fractures, as shown in Table 5.

**TABLE 1:**
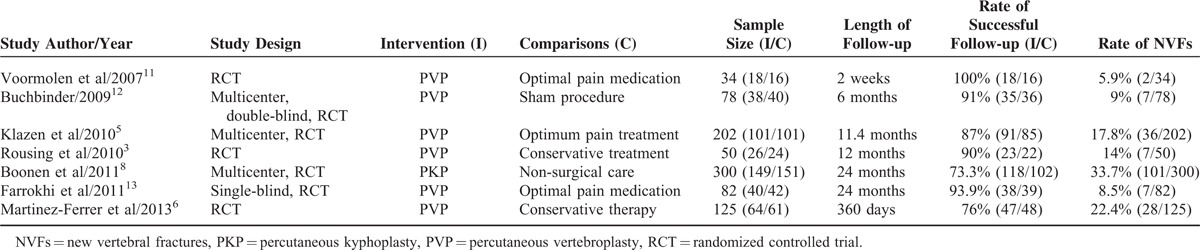
Summary of Study Characteristics Included in the Meta-Analysis

**TABLE 2:**
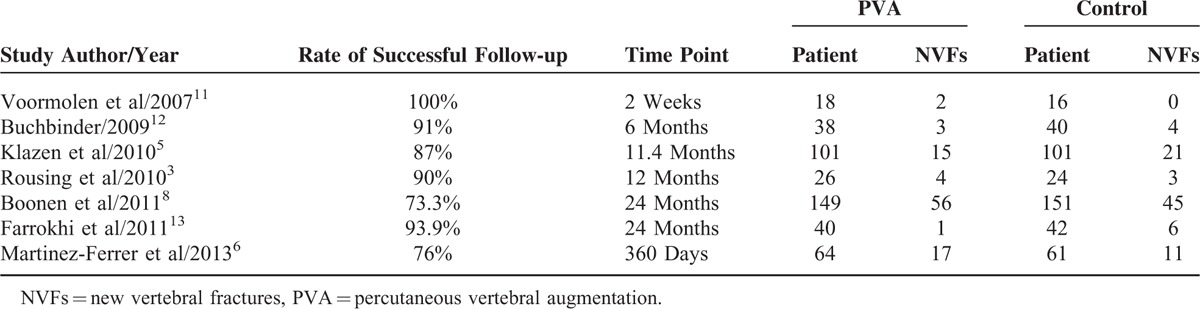
New Vertebral Fractures After Enrollment

**TABLE 3:**
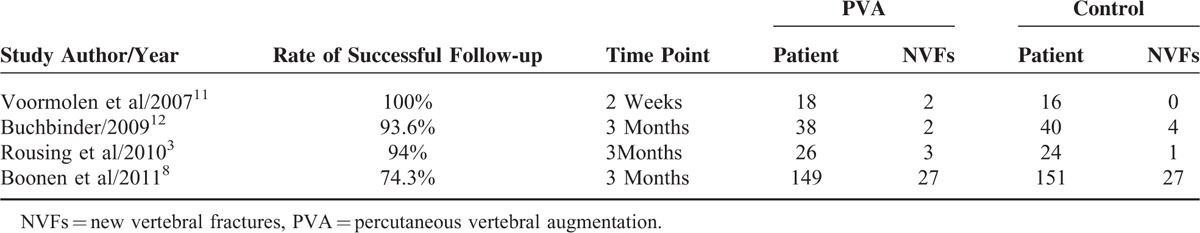
NVFs Within 3 Months

**TABLE 4:**
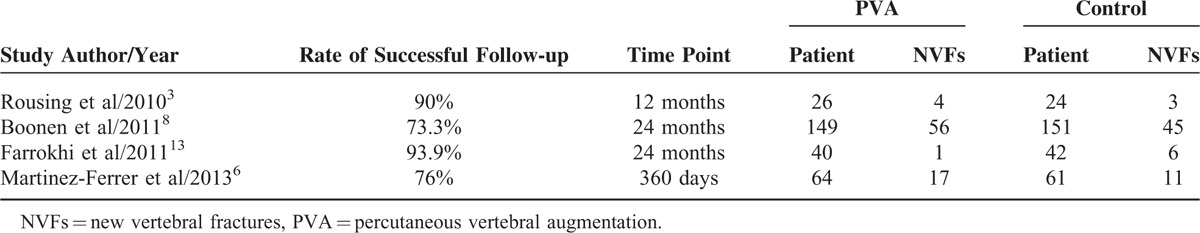
NVFs At Over 1 Year Follow-up

**TABLE 5:**
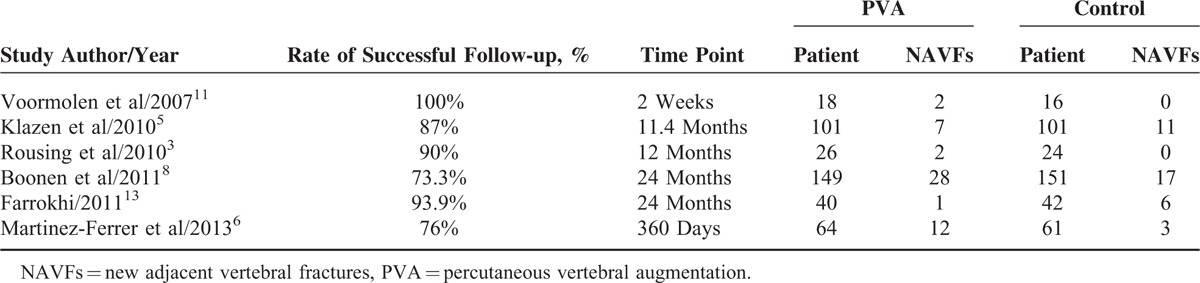
Incidence of Adjacent Fractures

### Literature Quality

**FIGURE 2:**
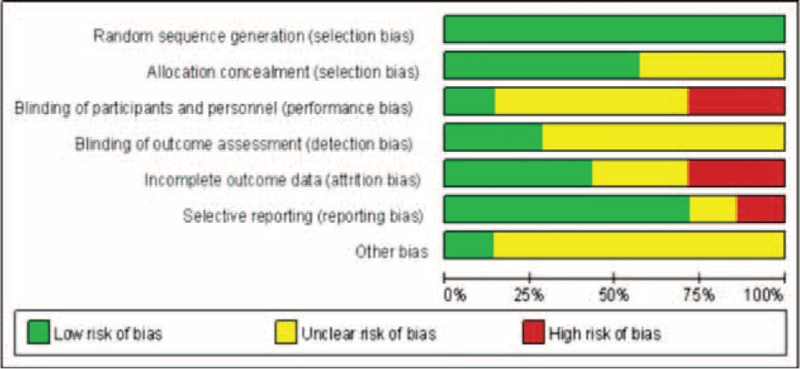
Risk of bias graph showing a review of the authors’ judgments about each risk of bias item presented as percentages across all included studies.

**FIGURE 3:**
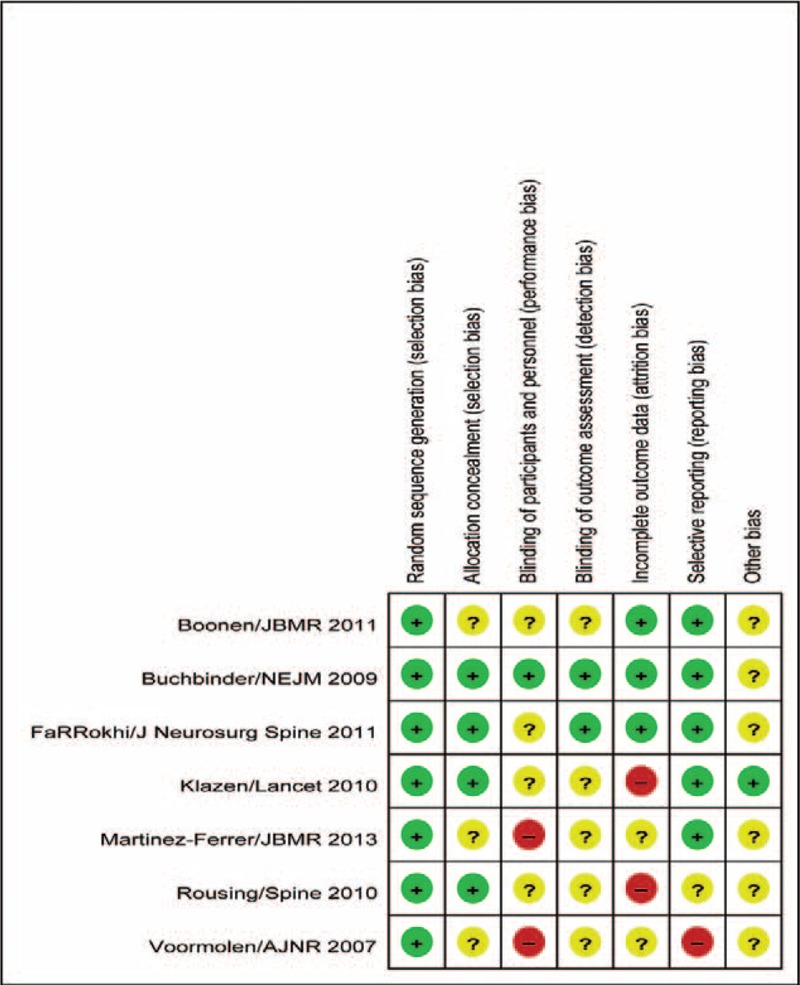
Risk of bias summary showing a review of the authors’ judgments about each risk of bias item for each included study.

In this study, the selected studies were assessed using the Cochrane Collaboration's tool for assessing risk of bias, as introduced in the Cochrane Handbook 5.3. The risk of bias was assessed from 6 perspectives. The selected studies all exhibited different levels of risk of bias. All 7 articles reported in detail the methods for randomized grouping, yet some did not describe the methods for hidden grouping, and only one adopted a double-blind method. Figures 2 and 3 illustrate the risk of bias existed in the selected studies.

### Research Results

**FIGURE 4:**
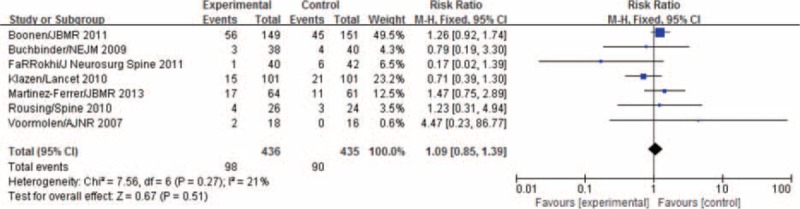
Forest plot of comparison: The Incidence of New Vertebral Fractures.

**FIGURE 5:**
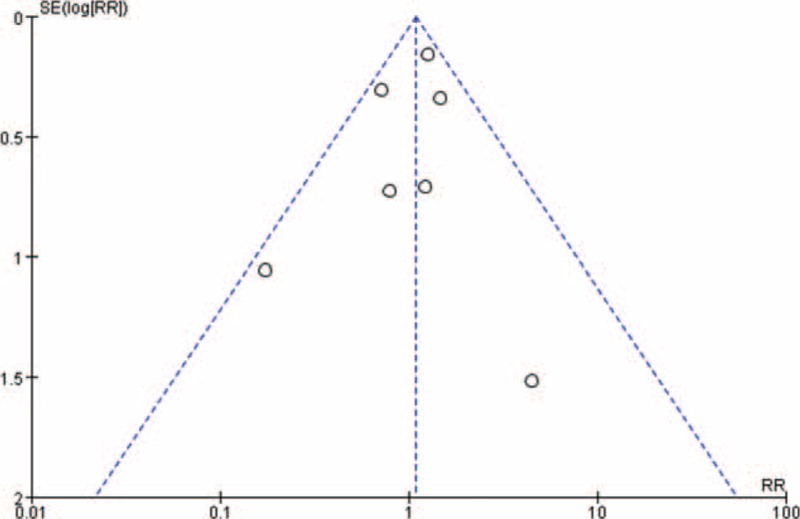
Funnel plot symmetrically distributed around the standardized mean difference.

The 7 selected studies all reported the incidences of new vertical fractures at the final follow-up for both groups of patients. Heterogeneous tests showed no heterogeneity among the studies (*P* = 0.27, *I*^2^ = 21%). From the fixed-effects model, the difference between the numbers of patients with new vertebral fractures in the 2 groups was not statistically significant (mean difference, MD = 1.09, 95% CI (0.85,1.39), as shown in Figure 4. No publication bias was observed; the funnel plots were symmetric about the mean standardized difference (Figure 5).

**FIGURE 6:**
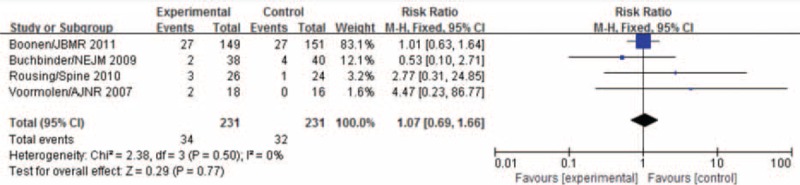
Meta-analysis of incidences of new vertebral fractures within 3 months after PVA and conservative treatment.

Four of the 7 studies reported the numbers of patients with new vertebral fractures occurred within 3 months after treatment. The heterogeneous test found no heterogeneity among the 4 studies (*P* = 0.50, *I*^2^ = 0%), and analysis with the fixed-effects model showed no statistical significance in the difference between the numbers of patients with new vertebral fractures in the 2 groups (MD = 1.07, 95% CI [0.69, 1.66]), as shown in Figure 6.

**FIGURE 7:**
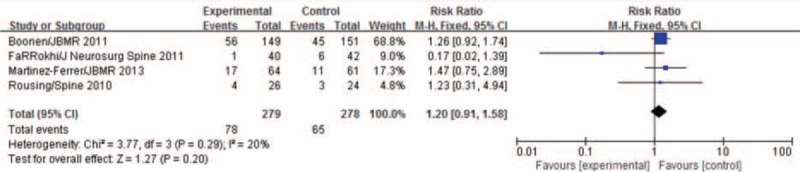
Meta-analysis of incidences of new vertebral fractures over one year after PVA and conservative treatment.

Four studies reported the numbers of patients with new vertebral fractures over 1 year of follow-up. There was no heterogeneity among the 4 studies (*P* = 0.29, *I*^2^ = 20%), and analysis with the fixed-effect models showed that the occurrence of new vertebral fractures over 1 year was not statistically significant (MD = 1.20, 95%CI [0.91,1.58]), as shown in Figure 7.

**FIGURE 8:**
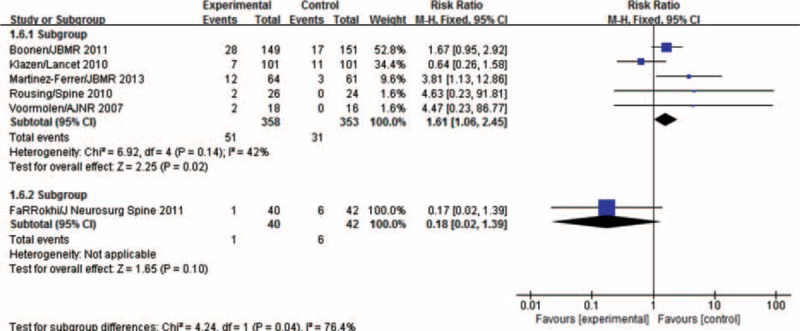
Meta-analysis of incidences of adjacent fractures after percutaneous vertebral augmentation and conservative treatment.

Six studies reported the numbers of patients with new fractures at vertebrae adjacent to the treated vertebrae. As there was heterogeneity among the 6 studies (*P* = 0.05, *I*^2^ = 54%), subgroup analysis was performed. Based on the origins of patients, the studies were divided into 2 groups. The European group included 5 studies^3,5,6,8,11^ with no heterogeneity (*P* = 0.14, *I*^2^ = 42%). The fixed-effects model showed that the incidence of new adjacent vertebral fractures among the PVA-treated patients is higher than that among the conservatively treated patients, and the difference is statistically significant (MD = 1.61, 95% CI [1.06,2.45]). The Asian group included 1 study,^13^ and there is no statistical significance within the group (MD = 0.18, 95% CI [0.02,1.39]). Figure 8 illustrates these results.

## DISCUSSION

Osteoporosis has become a common disease that threatens the health of older people, especially post-menopausal women, and vertebral fractures are the primary complication associated with osteoporosis. A study by Silverman et al^14^ showed that vertebral compression fractures occurred in 26% of women older than 50 years. Osteoporosis is one of the risk factors that results in bone fractures in the elderly, making the treatment of fractures more difficult. Therefore, improving the quality of fracture treatment and preventing new fractures has been the focus of the treatment of bone fractures among the elderly.

Vertebral fractures are the most common osteoporotic fractures and can occur repeatedly due to osteoporosis. Lindsay et al^15,16^ carried out a study of 381 patients with vertebral fractures, and reported that the incidence of new vertebral fractures within the subsequent year of fracture was 19.2%. They considered vertebral fractures to be an important predictive factor of bone fractures at other vertebrae or in other body parts. Further, they found that the presence of ≥1 vertebral fractures increases the risk of new vertebral fractures 5 times within 1 year.

PVA is a minimally invasive technique widely applied in recent years for the treatment of osteoporotic vertebral compressive fractures. PVA treatment helps stabilize the fracture, restores the mechanical strength of vertebrae, and prevents further compression of the vertebrae. Through PVA treatment, the pain of patient can be rapidly relieved, the quality of life greatly improved, and patients can begin moving again sooner. Generally, the short-term effects of PVA treatment are satisfactory. A study by Voormolen et al^11^ showed that pain relief and improvement of mobility, function, and stature after PVP was immediate and significantly better in the short term compared with conservative treatment. Van Meirhaeghe et al^9^ carried out a multicenter RCT of 300 patients to compare the efficacy and safety of PKP with nonsurgical management, and reported that PKP improves patient quality of life and pain averaged during 24 months.

However, PVA has been shown to increase the risk of new vertebral fractures, especially at vertebrae adjacent to the treated ones. Such concern has drawn wide attention. Lin et al^17^ classified the causes of new fractures into 2 categories, the biological causes of osteoporosis, and the biomechanical changes that result from PVA treatment. The progression of osteoporosis will result in further deterioration of bone quality, and consequentially lead to recurrent fractures. PVA also induces some chemical and physical changes in the vertebrae, which can lead to new fractures as a result of biomechanical changes.

At present, the effect of cement deposition into a fractured vertebra on the risk of subsequent fractures at other vertebrae remains unclear. There is theoretical concern that the diminishment of the compliance of one vertebra, as the result of cement injection, may place the remainder of the axial skeleton at greater risk of collapse. However, a different study^18^ found that the endplate deformation of fractured vertebrae under compressive load is reduced after PVA, restoring the nucleus pressure in adjacent intervertebral discs, and reduces the stress concentration in the posterior annulus. By restoring the normal load sharing, PVA treatment can potentially decrease the risk of recurrent fractures and new fractures at adjacent vertebrae.^19–21^

In this study, we attempted to include all related RCT studies, including the latest clinical reports. Combining the results of 7 RCT studies, we found that PVA treatment is not a significant cause of new vertebral fractures, even 24 months after treatment. However, 1 problem with this study is that the follow-up time is different across the 7 studies, varying from 3 months to 2 years; thus, a methodological difference may exist among the studies. Therefore, based on the follow-up time, we divided the studies into a short-term follow-up (3 months and shorter) group and long-term follow-up (1 year and longer) group. Further analysis of the 2 groups showed no significant differences between the incidences of new vertebral fractures within either group. This further confirmed the main point of this study that PVA does not significantly increase the incidence of new vertebral fractures compared with conservative treatments.

The location of the vertebrae with new fractures has also drawn great attention. Some scholars consider that PVA treatment significantly increases the mechanical load on vertebrae adjacent to the treated ones, thus increasing the incidence of fractures at adjacent vertebrae. Among the selected RCT studies, 6 studies reported the incidence of adjacent fractures. Considering the apparent heterogeneity among the studies, 2 subgroups were formed according to the origins of patients. The European group consists of 5 studies, which showed that the incidence of new adjacent fractures in patients receiving PVA treatments is significantly higher than that in patients who received conservative treatments. Meanwhile, the one study of the Asian group showed no statistical difference between the 2 groups. The difference between the 2 groups may be attributable to 2 reasons. First, in the study of Farrokhi et al, the overall incidence of new vertebral fractures is low; only 7 patients of 82 patients (8.5%) developed new fractures. The authors attributed the low incidence of new fractures to the use of unilateral puncture and the fact that some patients suffered from intravertebral vaccum and thereby needed less bone cement injection. Second, the study approach of Farrokhi et al allows the patients to cross over during the study's time span. During the 24-month follow-up period, 10 of the 42 patients (23.8%) in the conservative treatment group received PVA treatment. Therefore, a bias toward PVA treatment may exist in this study. In all the other 5 RCT studies, patients received PVA treatment suffered from higher incidence of fractures at vertebrae adjacent to the operated ones than the patients that received nonoperative treatments.

Some additional limitations exist in this study. Seven RCT experiments were selected, yet most of their methodologies were constrained. Four studies adopted multicenter methods, whereas the other 3 adopted single-center methods. Regarding the blinding, only 1 study adopted a double-blind method, 1 adopted a single-blind method, and none of the other 5 studies used a blinding method. Thus, the possibility of biased implementation may not be excluded. In addition, the fracture time, selection and exclusion criteria, treatments, follow-up times were different among the studies. Such differences affected the argumentation and reliability of this study to a certain extent. Furthermore, the relatively high dropout rates in some studies may also affect this meta-analysis. It is necessary to further design and implement large-sample, multi-center, randomized double-blind controlled trials to obtain more conclusive data.

Overall, the PVA treatment did not increase the incidence of new vertebral fractures in the studies selected. No significant differences were revealed between the incidences of new vertebral fractures after the 2 types of treatments, either in the short-term follow-up or in the long-term follow-up. Most of the studies selected in this study showed that PVA treatment increases the incidence of adjacent fractures, and that the incidence is higher than that after conservative treatments. However, one study showed no differences between the 2 types of treatments. More randomized double-blind controlled trials of a higher quality are needed to validate the question of incidence of adjacent fractures after PVA.

## References

[1] Trout AT Kallmes DF Kaufmann TJ New fractures after vertebroplasty: adjacent fractures occur significantly sooner. *AJNR Am J Neuroradiol* 2006; 27:217–223. 16418388 PMC7976057

[2] Klazen CA Venmans A de Vries J Percutaneous vertebroplasty is not a risk factor for new osteoporotic compression fractures: results from VERTOS II. *AJNR Am J Neuroradiol* 2010; 31:1447–1450. 20651016 10.3174/ajnr.A2148 PMC7966121

[3] Rousing R Hansen KL Andersen MO Twelve-months follow-up in forty-nine patients with acute/semiacute osteoporotic vertebral fractures treated conservatively or with percutaneous vertebroplasty: a clinical randomized study. *Spine* 2010; 35:478–482. 20190623 10.1097/BRS.0b013e3181b71bd1

[4] Rousing R Andersen MO Jespersen SM Percutaneous vertebroplasty compared to conservative treatment in patients with painful acute or subacute osteoporotic vertebral fractures: three-months follow-up in a clinical randomized study. *Spine* 2009; 34:1349–1354. 19478654 10.1097/BRS.0b013e3181a4e628

[5] Klazen CA Lohle PN de Vries J Vertebroplasty versus conservative treatment in acute osteoporotic vertebral compression fractures(Vertos II): an open-label randomised trial. *Lancet* 2010; 76:1085–1092. 20701962 10.1016/S0140-6736(10)60954-3

[6] Martinez-Ferrer A Blasco J Carrasco JL Risk factors for the development of vertebral fractures after percutaneous vertebroplasty. *J Bone Miner Res* 2013; 28:1821–1829. 23427068 10.1002/jbmr.1899

[7] Blasco J Martinez-Ferrer A Macho J Effect of vertebroplasty on pain relief, quality of life, and the incidence of new vertebral fractures: a 12-month randomized follow-up, controlled trial. *J Bone Miner Res* 2012; 27:1159–1166. 22513649 10.1002/jbmr.1564

[8] Boonen S Van Meirhaeghe J Bastian L Balloon kyphoplasty for the treatment of acute vertebral compression fractures: 2-year results from a randomized trial. *J Bone Miner Res* 2011; 26:1627–1637. 21337428 10.1002/jbmr.364

[9] Van Meirhaeghe J Bastian L Boonen S FREE investigators. A randomized trial of balloon kyphoplasty and nonsurgical management for treating acute vertebral compression fractures: vertebral body kyphosis correction and surgical parameters. *Spine* 2013; 38:971–983. 23446769 10.1097/BRS.0b013e31828e8e22 PMC3678891

[10] Wardlaw D Cummings SR Van Meirhaeghe J Efficacy and safety of balloon kyphoplasty compared with non-surgical care for vertebral compression fracture (FREE): a randomised controlled trial. *Lancet* 2009; 373:1016–1024. 19246088 10.1016/S0140-6736(09)60010-6

[11] Voormolen MH Mali WP Lohle PN Percutaneous vertebroplasty compared with optimal pain medication treatment: short-term clinical outcome of patients with subacute or chronic painful osteoporotic vertebral compression fractures. The VERTOS study. *AJNR Am J Neuroradiol* 2007; 28:555–560. 17353335 PMC7977842

[12] Buchbinder R Osborne RH Ebeling PR A randomized trial of vertebroplasty for painful osteoporotic vertebral fractures. *N Engl J Med* 2009; 361:557–568. 19657121 10.1056/NEJMoa0900429

[13] Farrokhi MR Alibai E Maghami Z Randomized controlled trial of percutaneous vertebroplasty versus optimal medical management for the relief of pain and disability in acute osteoporotic vertebral compression fractures. *J Neurosurg Spine* 2011; 14:561–569. 21375382 10.3171/2010.12.SPINE10286

[14] Silverman SL The clinical consequences of vertebral compression fracture. *Bone* 1992; 13:S27–S31. 1627411 10.1016/8756-3282(92)90193-z

[15] Lindsay R Silverman SL Cooper C Risk of new vertebral fracture in the year following a fracture. *JAMA* 2001; 285:320–323. 11176842 10.1001/jama.285.3.320

[16] Lindsay R Burge RT Strauss DM One year outcomes and costs following a vertebral fracture. *Osteoporos Int* 2005; 16:78–85. 15167988 10.1007/s00198-004-1646-x

[17] Lin H Bao LH Zhu XF Analysis of recurrent fracture of a new vertebral body after percutaneous vertebroplasty in patients with osteoporosis. *Orthopaed Surg* 2010; 2:119–123. 10.1111/j.1757-7861.2010.00074.x PMC6583431 22009926

[18] Hulme PA Ferguson SJ Boyd SK Determination of vertebral endplate deformation under load using micro-computed tomography. *J Biomech* 2008; 41:78–85. 17915227 10.1016/j.jbiomech.2007.07.018

[19] Luo J Adams MA Dolan P Vertebroplasty and kyphoplasty can restore normal spine mechanics following osteoporotic vertebral fracture. *J Osteoporos* 2010; 20:1–9. 10.4061/2010/729257 PMC2957176 20981329

[20] Luo J Skrzypiec DM Pollintine P Mechanical efficacy of vertebroplasty: influence of cement type, BMD, fracture severity, and disc degeneration. *Bone* 2007; 40:1110–1119. 17229596 10.1016/j.bone.2006.11.021

[21] Farooq N Park JC Pollintine P Can vertebroplasty restore normal load-bearing to fractured vertebrae? *Spine* 2005; 30:1723–1730. 16094273 10.1097/01.brs.0000171906.01906.07

